# Supplementary material for: Reconciling Mining with the Conservation of Cave Biodiversity: A Quantitative Baseline to Help Establish Conservation Priorities
Source: PLoS One. 2016 Dec 20;11(12):e0168348. doi: 10.1371/journal.pone.0168348 (PMC5173368; doi:10.1371/journal.pone.0168348)
Supplement: S1 Dataset — (ZIP) [file pone.0168348.s002.zip › Taxa/Serra Norte/SN_2013/N5S/Lista N5S-29.pdf]

CAVIDADE N5S-0029

Classe	Ordem	Fam/Outros	Gên/Outros	Espécie	S-U	U-S	Zon.
Arachnida	Araneae	Araneidae		jovem		X	E
Arachnida	Araneae	Ctenidae		jovem		2	E
Arachnida	Araneae	Drymusidae		jovem		X	E
Arachnida	Araneae	Mysmenidae		jovem	X		E
Arachnida	Araneae	Pholcidae	*Mesabolivar*	*eberhard*	X	X	E
Arachnida	Araneae	Theridiidae	*Achaearanea*	sp.1		X	E
Arachnida	Araneae	Theridiosomatidae		jovem	X	X	E
Arachnida	Opiliones	Sclerosomatidae	*Prionostema*	sp.1		X	E
Arachnida	Pseudoscorpiones	Chernetidae	*Spelaeochernes*	sp.1	X	X	E
Entognatha	Collembola	Paronellidae		sp.1	X	X	E
Entognatha	Diplura	Japygidae		sp.3		X	E
Insecta	Blattodea			jovem		2	E
Insecta	Coleoptera	Curculionidae	Scolytinae	sp.2		X	E
Insecta	Coleoptera	Staphylinidae		sp.28		X	E
Insecta	Diptera	Cecidomyiidae	Cecidomyiinae	sp.	X		E
Insecta	Diptera	Ceratopogonidae		jovem	X		E
Insecta	Diptera	Chironomidae		sp.		X	E
Insecta	Diptera	Phoridae	Metopininae	sp.	X		E
Insecta	Diptera	Psychodidae	*Sciopemyia*	*sordellii*	X	X	E
Insecta	Diptera	Tipulidae	Tipulinae	sp.	X		E
Insecta	Heteroptera	Coreidae		jovem	X		E
Insecta	Heteroptera	Miridae		jovem	X		E
Insecta	Heteroptera	Reduviidae	Reduviinae	jovem	2		E
Insecta	Homoptera	Cixiidae		sp.5	X	X	E
Insecta	Hymenoptera	Formicidae	*Acropyga*	sp.1	X	X	E
Insecta	Hymenoptera	Formicidae	*Apterostigma*	sp.1		X	E
Insecta	Hymenoptera	Formicidae	*Pachycondyla*	*striata*		1	E
Insecta	Hymenoptera	Vespidae		sp.6		1	E
Insecta	Lepidoptera	Gelechioidea		sp.4		X	E
Insecta	Lepidoptera	Noctuoidea		sp.8	3	1	E
Insecta	Neuroptera	Myrmeleontidae		jovem		2	E
Insecta	Psocoptera	Ptiloneuridae	*Ptiloneura*	sp.1	X	X	E
Gastropoda	Pulmonata	Bulimulidae	*Naesiotus*	sp.	X		E
Malacostraca	Isopoda	Dubioniscidae		sp.1		X	E
Amphibia	Anura	Strabomantidae	*Pristimantis*	cf. *fenestratus*		1	E
